# Sanitary Science and Sanitary Legislation

**Published:** 1855-09

**Authors:** 


### JOURNAL OF PUBLIC HEALTH.

SEPTEMBER 1855.

### SANITARY SCIENCE AND SANITARY LEGISLATION.

THE great events of the last two years, despite the anxieties they have excited in this country, have not been without their moral and practical value. The most painful pictures in our late and still continuing struggle have taught a solemn lesson; and England, rising now from the temporary gloom into which she has been thrown, looks round again, subdued in some sense, but feeling in this very subjection an increase of strength, based on an improved knowledge, a more vigilant foresight, a keener appreciation of the weak points in her own character, and a more determined desire to be guided in the future by the teaching of the past. The effects of this change on the views and opinions of the nation have manifested themselves in no department of knowledge more than in that which relates to sanitary science. It is universally felt, that to a spirit either of ignorance or indifference on this subject the great living sacrifice of the brave men who, unhurt by the enemy's fire, died in the East last winter, is mainly due; and this fact, coupled with the fear of the cholera plague, which has recently paid so many and fearful visits, has at length urged the people to consider seriously the meaning of health and disease, and to listen to the useful sanitary lessons, which many earnest men have for long years past tried with too little success to explain and teach.

The sanitary movement, therefore, is now what is commonly called the leading subject of the day. The daily and weekly press is full of it; parliamentary debaters even are anxious to discuss it; the medical profession, in its usual spirit of true nobility, is foremost in desiring to forward it, albeit its success is in direct opposition to pecuniary interests of a professional kind; many of the clergy are becoming charged with the idea that it is their mission to work and preach in so good a cause; and literary and mechanics' institutions are throwing open their lecture rooms freely for the use of the sanitary teacher.

These are all refreshing and hopeful signs; and if this popular feeling in behalf of the health movement be but judiciously cultivated, the results will become, in the end, of an importance which few can at this moment realise.

But intended reforms, to be safe, must be gradual—must be founded on actual knowledge, and must be understood by the masses in the order they are set forth. This statement, which holds good in a general sense, is specially true in relation to sanitary inquiries; for so intimately is each step in the art of preserving health mixed up with social questions of various kinds, that a few false moves, or impracticable and perhaps unscientific attempts hastily made by amateur sanitarians, for the purpose of doing something, may merely do harm ultimately to the cause they were intended to support.

We make these observations because it is obvious to us, and to many others who, like ourselves, are by education and occupation specially devoted to the pursuit of sanitary knowledge, that the efforts, which at the present are being made by the parliament and by many public bodies, are undertaken without regard to system, and without any definite or comprehensive object. The elements of the sanitary question are few in number, but they lie much deeper than is generally supposed, and many recent and notable health measures are but temporary in their character, and scarcely calculated to affect the great questions at issue, either for evil or for good.

There is a strong feeling abroad at this moment that legislative enactments are capable of doing service in the preservation of health, and the suppression of disease. We do not deny that a decision in a law court may occasionally check or remove some real or supposed cause of disease; but we doubt the correctness of the principles of coercion, and we more than doubt the general competency of the men upon whom will devolve the duty of inflicting fines and penalties on those adjudged guilty of breaking some new health law.

The labours of the true sanitary reformer lie, we believe, in but four directions.

1st. In an endeavour to understand simply the nature of diseases, their alliances, their true distinctive characters, the modifications to which the body is subjected under the influence of diseased action, and the chemical or physical measures best adapted for removal and prevention of disease. Labours of this kind require the highest order of medical intellect; they are altogether removed from the business of the mere politician, and they imply in him who prosecutes them a mind fully charged with all the modern doctrines of chemistry, physics, and the laws of life. These labours tend to lead the mind from effects back to causes.

2nd. In an endeavour to seek out primarily the causes of diseases, irrespective of the symptoms and the other details involved in the consideration of the diseases themselves. Efforts in this line of research should embrace observations conducted on a large scale, and bearing on the effects of locality, climate, season, meteorology, contagion, habit, diet, and occupation, in giving rise to distinctive types of disease.

3rd. In striving to make the vast stores of information already acquired in regard to the two forms of inquiry above noted accessible to all classes of society, by having them scientifically popularised and diligently taught, especially amongst the child population.

4th. In giving free scope and encouragement to those mechanical arts which tend to improve the beauty and convevience of towns and cities, to lessen muscular labour, and to increase the comforts of the poor man's home; and in instituting such an elevated class of amusements and occupations for leisure moments, as shall make the heart more happy and the mind less animal.

In these offerings to reason and knowledge lies the true and one elixir vitæ, possessing in truth all the charms which the quacksalver's bill claims for the quacksalver's pill.

In the prosecution of the few and simple principles, thus sketched out, is embodied, we believe, the beginning and the end of sanitary science. These ignored, all else is mere practical dilettanteism—a playing with the details of principles, while the principles themselves are undeveloped —a common error. Right honourable and honourable gentlemen may discourse in the senate house, and sit on committees of inquiry, and publish tons weight of blue books, and preside over Boards of Health, and invest magistrates with new powers; but the mortality tables of the Registrar-General will still tell how little can be done until a new impulse is given bearing on the leading measures which we have endeavoured, however feebly, to define and point out.

There are some diseases which seem to stand forth independent altogether of what are usually understood as pernicious influences—diseases which have a specific cause of which we know nothing whatever, and cannot even form an idea. And there are other diseases, the propagation of which is as easily traceable as is the generation of the living world. If legislation must do something (as it ought to do), why should it not direct inquiry in reference to this distinction? and when it has made out a few facts regarding those diseases, the propagation of which is understood, why should it not make a trial to lessen or extinguish them? There is a disease of diseases which a mock morality makes it a very crime to name, but which leaves its base imprint more or less marked on every hundredth babe at least born in this great metropolis, and which engrafts a host of maladies on half our Saxon race. This of all diseases is most preventible by coercive legislation, if such legislation can do anything whatever. But it cannot. It cannot stop small-pox, though it has Jenner's discovery in its right hand. In short, in relation to sanitary science the legislature can do but one thing—namely, promote scientific education. But if it would only do this, it would speedily do all.

---

